# Experimental Effects of Acute Exercise in Attenuating Memory Interference: Considerations by Biological Sex

**DOI:** 10.3390/medicina55070331

**Published:** 2019-07-02

**Authors:** Lauren Johnson, Lindsay Crawford, Liye Zou, Paul D. Loprinzi

**Affiliations:** 1 Exercise & Memory Laboratory, Department of Health, Exercise Science and Recreation Management, The University of Mississippi, Oxford, MS 38677, USA; 2 Shenzhen Key Laboratory of Affective and Social Cognitive Science, College of Psychology and Sociology, Shenzhen University, Shenzhen 518060, China

**Keywords:** cognition, encoding, learning, memory, physical activity

## Abstract

*Background and Objectives:* The objective of this experiment was to evaluate the effects of acute exercise on memory interference and determine if this potential relationship is moderated by sex. *Materials and Methods:* A randomized controlled experiment was conducted (N = 40), involving young adult males (n = 20) and females (n = 20) completing two counterbalanced visits (exercise and no exercise). The exercise visit involved an acute (15 min), moderate-intensity bout of treadmill exercise, while the control visit involved a time-matched seated task. Memory interference, including both proactive interference and retroactive interference, involved the completion of a multi-trial memory task. *Results:* In a factorial ANOVA with the outcome being List B, there was a main effect for condition (F(1,38) = 5.75, P = 0.02, n^2^_p_ = 0.13), but there was no main effect for sex (F(1,38) = 1.39, P = 0.24, n^2^_p_ = 0.04) or sex by condition interaction (F(1,38) = 1.44, P = 0.23, n^2^_p_ = 0.04). *Conclusion:* In conclusion, acute moderate-intensity exercise was effective in attenuating a proactive memory interference effect. This effect was not moderated by biological sex.

## 1. Introduction

Within the field of neurophysiology, emerging research demonstrates that acute exercise (single bout of exercise) may improve short-term and long-term episodic memory function (retrospective recall of an event in a spatiotemporal context) [1,2,3]; we have discussed the mechanistic details of this effect thoroughly elsewhere [4,5,6,7]. A novel line of inquiry, however, is whether acute exercise can attenuate a memory interference effect. Memory interference is often classified into proactive and retroactive memory interference. Proactive interference involves a preceding stimulus interfering with the acquisition and retention of a subsequent stimuli. For example, learning words from List A may interfere with the recall of learning a second list (i.e., List B). Retroactive interference, however, involves a subsequent stimulus interfering with the recall of a previously encoded stimuli. For example, after learning words from List A and then List B, the encoding of List B may interfere with the subsequent retrieval of List A.

In addition to evaluating the effects of acute exercise on episodic memory function, our lab is interested in examining whether acute exercise can attenuate a cognitive (vs. procedural [8]) memory interference effect. At the time of this writing, only four published experiments have evaluated this possibility, which have come from our laboratory [9,10,11]. In the first experiment on this topic, we employed a six group, between-subject study. In this study, three groups focused on proactive interference and three groups on retroactive interference. Within each of the three proactive interference groups, one group exercised and also engaged in a memory task designed to induce proactive interference, another group went through the proactive interference paradigm but did not exercise, and lastly, another group did not exercise and completed a memory task without a proactive interference protocol. A similar three-group approach was implemented for the three retroactive interference groups. In this six-group experimental study, we provided some suggestive evidence, albeit weak, that acute moderate-intensity exercise may attenuate a retroactive interference effect [11]. In our second experiment, we employed a multi-trial verbal learning task that involved learning two separate word lists (Lists A and B). Within this study, we implemented groups that either engaged in a moderate-intensity or high-intensity bout of exercise shortly before the learning task. In this between-subject experiment, we provided suggestive evidence, again albeit weak, that acute exercise, particularly high-intensity exercise, attenuated proactive memory interference [9]. Lastly, employing a within-subject design, while also utilizing a multi-trial learning task, we provided some evidence suggesting that acute moderate-intensity exercise can attenuate a proactive interference effect [10]. Most recently, we implemented an AB/AC paradigm to evaluate memory interference, and although acute high-intensity exercise enhanced episodic memory function, acute exercise did not attenuate memory interference [12].

The present experiment extends these four previous experiments by specifically evaluating whether there is a sex-specific effect of acute exercise on attenuating a memory interference effect. As we have thoroughly detailed elsewhere [13], such an effect is plausible, as there is a clear sex-specific effect on memory function. That is, females tend to outperform males on nearly all memory tasks, with the exception of spatial-based memory tasks [13]. However, males may have a greater exercise-induced neurotropic response that may mediate improvements in memory function [13]. To extend this emerging line of inquiry (i.e., exercise and memory interference), and to address this potential sex-specific effect, the purpose of this study was to evaluate whether the potential attenuation effect of acute exercise on memory interference is moderated by biological sex. We had no specific hypothesis [14] on a potential sex-specific effect, given that biological sex appears to have a differential effect on memory function and the exercise-induced response to molecular mediators of memory function.

## 2. Methods

### 2.1. Study Design

A randomized controlled intervention was employed. Both males and females completed two counterbalanced laboratory visits, with one visit involving a 15 min bout of exercise prior to the memory task. The control visit engaged in a time-matched seated task. Each within-subject visit occurred around the same time of day (±2 h) and occurred at least 24 h after the first visit. This study was approved by the University of Mississippi’s ethics committee (#18-123; approved on 5 June 2018). All participants provided written consent prior to study participation.

### 2.2. Participants

In total, 40 participants were recruited (20 males and 20 females). This is based from a power analysis indicating a sample size of 20 would be needed for sufficient power (d, 0.90; two-tailed α error probability, 0.05; 1-β error probability, 0.80; allocation ratio, 1). Our evaluated sample of 40 (n = 20 per group) is in alignment with (and double the size of some related studies [10,15]) other related experiments [3,15,16,17]. Recruitment occurred via a convenience-based, non-probability sampling approach (classroom announcement and word-of-mouth). Participants included undergraduate and graduate students between the ages of 18 and 35 years. This paper is a secondary analysis of a previous paper that focused on the individual learning trials (but not proactive or retroactive memory interference) [18].

Additionally, and identical to other studies [19], participants were excluded if they:

Self-reported as a daily smoker [20,21].
Self-reported being pregnant [22].
Exercised within 5 h of testing [23].
Consumed caffeine within 3 h of testing [24].
Had a concussion or head trauma within the past 30 days [25].
Took marijuana or other illegal drugs within the past 30 days [26].
Were considered a daily alcohol user (>30 drinks/month for women; and >60 drinks/month for men) [27].

### 2.3. Exercise Protocol

The exercise bout involved exercising on a treadmill for 15 min. Participants exercised at approximately 70% of their estimated heart rate max (220-age), which corresponds with moderate-intensity exercise [28]. We have previously shown that this intensity is sufficient in enhancing memory function [1,2,3,15]. This target heart rate was achieved by manipulating the speed/incline during the exercise bout to stay within 5 beats per minute of the target heart rate.

Immediately after the bout of exercise, participants rested in a seated position for 5 min. During this resting period, they played an on-line game of Sudoku (described below) to prevent boredom. After this resting period, they commenced the memory assessment, as described below. We have experimental evidence that playing Sudoku does not prime or enhance memory function [29].

### 2.4. Control Protocol

For the control visit, and similar to other studies [30], participants completed a medium-level, on-line administered, Sudoku puzzle for 20 min. The website for this puzzle is located here: https://www.websudoku.com/

### 2.5. Memory Assessment

**Figure 1:**
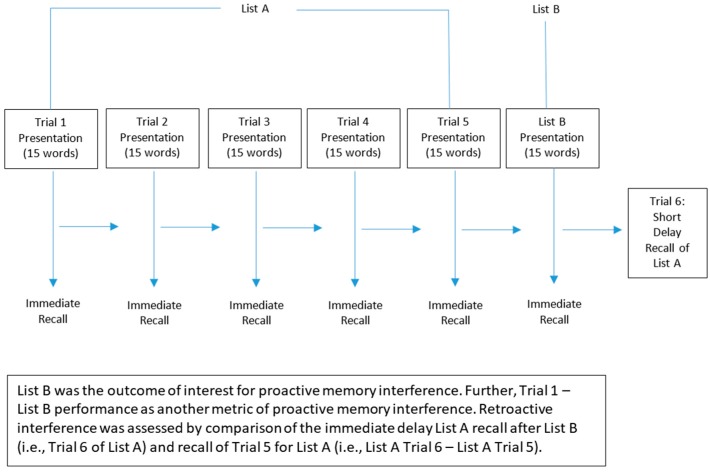
Schematic of the memory assessment protocol.

Identical to our related experimental work [3,15,17], memory was assessed using the standardized Rey Auditory Verbal Learning Test (RAVLT) [31]. Participants listened to and immediately recalled a recording of a list of 15 words (List A) five times in a row (Trials 1–5). Each word list was recorded at a rate of approximately 1 word per second. Participants then were asked to listen to and immediately recall a list of 15 new words (List B). Following this, participants recalled as many words from List A as possible. See Figure 1 for a schematic of this protocol.

Identical to our other experimental work on memory interference [9,10], performance (number of words correctly recalled) on List B was the outcome of interest for proactive memory interference. Further, we also evaluated Trial 1 – List B (i.e., number of words recalled for Trial 1 minus the number of words recalled for List B) performance as another metric of proactive memory interference. Retroactive interference was assessed by comparison of the immediate delay List A recall after List B (i.e., Trial 6 of List A) and recall of Trial 5 for List A (i.e., List A Trial 6 – List A Trial 5). Thus, outcome measures included the number of words recalled for Trial 1 (List A), Trial 5 (List A), Trial 6 (List A), and List B.

### 2.6. Additional Assessments

Various demographic (e.g., body mass index (BMI)) and behavioral (i.e., habitual physical activity) assessments were also assessed. As a measure of habitual physical activity behavior, participants completed the Physical Activity Vital Signs Questionnaire to evaluate time spent per week in moderate-to-vigorous physical activity (MVPA) [32]. This MVPA questionnaire asks participants two questions, namely the number of days per week they engage in MVPA and the amount of time per day they engage in MVPA. Specifically, they were asked, “*On average, how many days per week do you engage in moderate to strenuous exercise*” and “*On average, how many minutes do you engage in exercise at this level*”? Weekly engagement in MVPA was calculated based on the product of these two responses (i.e., days*minutes). Height/weight (BMI; kg/m^2^) were measured to provide anthropometric characteristics of the sample. Lastly, before and at the end of the exercise and control conditions, heart rate (chest-strapped Polar monitor, F1 model) was assessed.

### 2.7. Statistical Analysis

All statistical analyses were computed in JASP (v. 0.9.1). A 2 (sex; male vs. female) × 2 (condition; exercise vs. control) repeated measures ANOVA was computed to evaluate whether biological sex moderated the potential effects of acute exercise on memory interference. Bonferroni-corrected post-hoc tests were employed when main or interaction effects occurred. Statistical significance was set at an alpha of 0.05.

## 3. Results

***Participant Characteristics***. Table 1 displays the demographic and behavioral characteristics of the sample. Participants, on average, were 20.8 (0.9) years of age. There were no significant BMI (P = 0.98) or race-ethnicity (P = 0.11) differences across the two sexes. However, males (224 min/week) were significantly (P = 0.006) more active than females (107.4 min/week of moderate-to-vigorous physical activity).

***Physiological Responses from Exercise***. Table 2 displays the exercise responses. Heart rate was significantly (P < 0.001) higher at the endpoint of exercise when compared to baseline for both males and females. That is, across both sexes, heart rate increased by approximately 45 beats per minute from resting to the end of the exercise bout.

**Figure 2:**
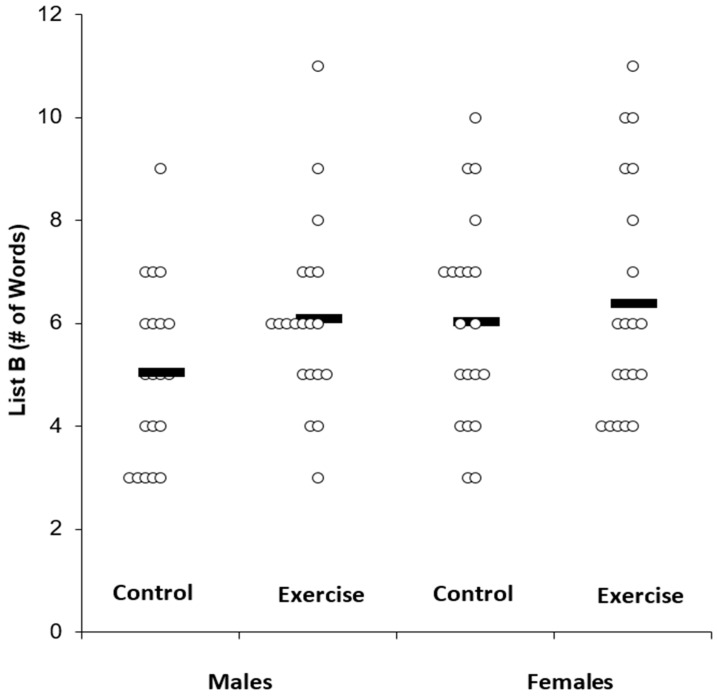
List B memory scores across condition. Solid bar represents the group average.

**Figure 3:**
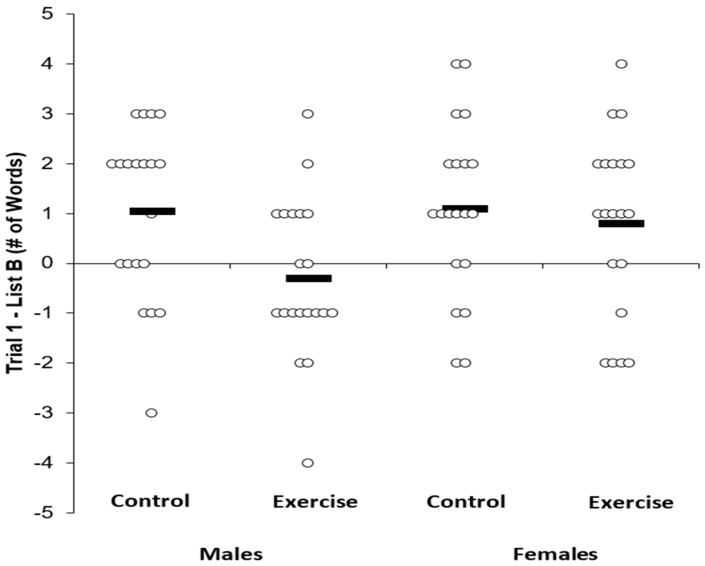
Trial 1 - list B memory scores across condition and sex. Solid bar represents the group average.

***Proactive Memory Interference***. Table 3 displays the memory scores across the experimental conditions and by sex. Individual data for List B is shown in Figure 2, whereas individual data for the Trial 1 – List B results are shown in Figure 3.

In a 2 × 2 ANOVA with the outcome being List B, there was a main effect for condition (F(1,38) = 5.75, P = 0.02, n^2^_p_ = 0.13), but there was no main effect for sex (F(1,38) = 1.39, P = 0.24, n^2^_p_ = 0.04) and no sex by condition interaction (F(1,38) = 1.44, P = 0.23, n^2^_p_ = 0.04). Post-hoc tests indicated a Bonferroni-corrected statistically significant difference in List B between exercise (6.2 ± 2.0) and control conditions (5.5 ± 1.9) (t = 2.39, P = 0.02).

Similarly, in a 2 × 2 ANOVA with the outcome being Trial 1 - List B, there was no main effect for sex (F(1,38) = 1.90, P = 0.17, n^2^_p_ = 0.05) and no sex by condition interaction (F(1,38) = 2.26, P = 0.14, n^2^_p_ = 0.06), but there was a main effect for condition (F(1,38) = 5.60, P = 0.02, n^2^_p_ = 0.12), and this main effect was not altered when controlling for MVPA (F(1,38) = 4.29, P = 0.04, n^2^_p_ = 0.11). Post-hoc tests indicated a Bonferroni-corrected statistically significant difference in Trial 1 - List B between exercise (0.25 ± 1.78) and control conditions (1.08 ± 1.7) (t = 2.32, P = 0.02).

Although we did not observe a main effect for sex (F(1,38) = 1.90, P = 0.17, n^2^_p_ = 0.05) or condition by sex interaction (F(1,38) = 2.26, P = 0.14, n^2^_p_ = 0.06) for Trial 1 – List B, in a 2 (male, female) × 2 (Trial 1 exercise, Trial 1 control) ANOVA, there was a main effect for sex for Trial 1 (F(1,38) = 2.26, P = 0.01, n^2^_p_ = 0.15).

***Retroactive Memory Interference***. Table 3 also shows the retroactive memory scores (Trial 6; Trial 5; and Trial 6 – Trial 5).

In a 2 × 2 ANOVA with the outcome being Trial 6 – Trial 5, there was no main effect for condition (F(1,38) = 0.16, P = 0.69, n^2^_p_ = 0.004), no main effect for sex (F(1,38) = 1.43, P = 0.23, n^2^_p_ = 0.04), and no sex by condition interaction (F(1,38) = 0.31, P = 0.58, n^2^_p_ = 0.008).

## 4. Discussion

The motivation for this study was twofold: (1) very limited research has evaluated the effects of exercise on memory interference and (2) no study has evaluated whether there is a sex-specific effect of this potential relationship. As such, the purpose of this study was to evaluate the effects of acute exercise on attenuating a memory interference effect and whether biological sex moderates this potential relationship. The present study provides experimental evidence that acute, moderate-intensity, short-duration exercise, attenuates proactive memory interference. We did not, however, observe evidence that biological sex moderates this effect. In alignment with other work [13], we did, however, demonstrate a main effect for sex for Trial 1, suggesting a female superior effect on short-term memory.

Regarding our observed association of acute exercise in reducing a proactive interference effect, recent work demonstrates that the prefrontal cortex, which is activated via acute exercise [33], plays an important role in minimizing a proactive interference effect via encoding and retrieval-based mechanisms. For example, the prefrontal cortex facilitates pattern separation in the dentate gyrus, and in turn, helps minimize a proactive interference effect [34]. Notably, BDNF appears to be necessary for the consolidation of pattern-separated memories in the dentate gyrus [35]. Given the very short period between the learning trials, it is likely that BDNF did not play any role in memory consolidation in the present study. However, in addition to memory consolidation, BDNF may influence memory in other ways, such as facilitating neurotransmitter release [36], which, ultimately, may facilitate the acquisition of the memory trace.

In the present experiment, we observed evidence that acute exercise attenuated a proactive memory interference effect but did not attenuate a retroactive interference effect. Typically, proactive interference is thought to be related to the integrity of executive function, with executive control processes thought to minimize proactive interference effects [37]. The prefrontal cortex plays a critical role in executive control and acute exercise has been shown to improve executive control processes [38,39]. Retroactive interference, on the other hand, may be more susceptible to the integrity of the memory trace and under influence by consolidation processes [40]. Perhaps our relatively short delay period (i.e., the length it took to complete List B) was not long enough to fully consolidate the memory (List A), and thus, prevented an exercise-induced attenuation effect of retroactive memory interference. Although speculative, perhaps acute exercise had a greater effect on proactive interference, as opposed to retroactive interference, via its influence on executive control (via inhibiting List A) and attention-related influences on memory encoding (particularly List B). This is an area ripe for future research. We have previously discussed the importance of exercise temporality on episodic memory function [3,15,17,41,42] and maybe it also plays an important role in retroactive interference. For example, if retrospective interference is under consolidation-related processes, then if the acute bout of exercise occurs during the consolidation phase, this may help to attenuate a retroactive interference effect. Relatedly, we have recently shown that when exercise occurs during the consolidation phase, this helps to stabilize memory function [43].

This work has important clinical implications. Memory function plays a critical role in daily functioning. Memory interference has a profound effect in influencing memory retention, and as such, identifying factors that attenuate memory interference is a worthwhile endeavor. The present experiment provides suggestive evidence that an acute bout of moderate-intensity exercise may help to attenuate a proactive memory interference effect. Future work should evaluate whether chronic exercise has a similar effect.

## 5. Conclusions

In conclusion, our experimental results provide suggestive evidence that an acute bout of moderate-intensity exercise can attenuate a proactive memory interference effect. This effect does not appear to be driven by biological sex, despite females demonstrating greater short-term memory when compared to males.

## Figures and Tables

**Table 1:** Characteristics of the sample.

Variable	Males (n = 20)	Females (n = 20)	P-Value
Age, mean years	20.95 (1.1)	20.65 (0.81)	0.34
Non-Hispanic white, %	75.0	95.0	0.11
BMI, mean kg/m^2^	26.20 (3.4)	26.23 (4.8)	0.98
MVPA, mean minutes/week	224.0 (161.0)	107.4 (72.1)	0.006

BMI, body mass index; MVPA, moderate-to-vigorous physical activity; values in parentheses are standard deviations.

**Table 2:** Heart rate responses across the conditions.

	Males		Females	
**Variable**	**Exercise**	**Control**	**Exercise**	**Control**
Baseline heart rate, mean bpm	84.55 (13.11)	72.65 (14.2)	93.75 (11.3)	83.4 (16.0)
Endpoint heart rate, mean bpm	130.6 (10.2)	76.25 (16.3)	136.0 (11.3)	87.25 (18.4)

Values in parentheses are standard deviations; bpm, beats per minute.

**Table 3:** Memory scores across the experimental conditions by sex.

	Males		Females	
**Variable**	**Exercise**	**Control**	**Exercise**	**Control**
Trial 1, mean # words recalled	5.80 (1.7)	6.10 (1.6)	7.20 (2.0)	7.15 (2.0)
List B, mean # words recalled	6.10 (1.8)	5.05 (1.7)	6.40 (2.3)	6.05 (2.0)
Trial 1 – List B, mean # words recalled	−0.3 (1.5)	1.05 (1.7)	0.80 (2.5)	1.10 (1.7)
Trial 5, mean # words recalled	12.20 (1.9)	12.35 (1.9)	13.00 (2.6)	12.70 (1.4)
Trial 6, mean # words recalled	10.70 (2.4)	10.55 (2.4)	11.85 (2.6)	11.60 (2.3)
Trial 6 – Trial 5, mean # words recalled	−1.50 (1.8)	−1.80 (1.5)	−1.15 (1.7)	−1.10 (1.6)

Values in parentheses are standard deviations.
